# Risk of infection in the first year of life in preterm children: An Austrian observational study

**DOI:** 10.1371/journal.pone.0224766

**Published:** 2019-12-09

**Authors:** Laura Steiner, Susanne C. Diesner, Peter Voitl

**Affiliations:** 1 First Vienna Pediatric Medical Center, Vienna, Austria; 2 Sigmund Freud University Vienna, Vienna, Austria; Univesity of Iowa, UNITED STATES

## Abstract

Newborns, especially preterm infants, have an immature immune system, which, in combination with the required medical interventions necessary for keeping the neonate alive may lead to an increased risk of infection. Even after reaching stability and adapting to the environment, preterm infants have adverse prognoses regarding infections and long-term outcomes compared to their full-term counterparts. The objective of this study was to research differences in the number and severity of infections between preterm and full-term infants during their first year of life. To answer this question, a monocentric prospective study was conducted in a pediatric practice in Vienna, including 71 full-term infants and 72 preterm infants who were observed during their first year of life regarding occurring infections. In respective samples, there was a significantly higher total number of infections in preterm (mean 6.01 ± 3.90) compared to full-term infants (3.85 ± 1.72) during the observation period of one year. Particularly the count of respiratory and severe infections was considerably higher in preterm infants. Otorhinolaryngeal infections were the most frequent of all types of infections in both groups. The pregnancy period, number of siblings, and length of the postnatal hospital stay, were observed as significantly influencing factors which affected the total number of infections. The group of early term infants (37+0 weeks to 38+6) was not significantly different to late term babies (>39+0). The acquired knowledge about the increased risk of infections should lead to a more extensive care for preterm infants, with the objective of reducing the rates of complications, morbidity and mortality in this vulnerable age group in the future.

## Background

A premature birth is by definition a birth taking place before the 37th week of gestation (WOG), with further classification into late (32–37 WOG), very (28–32 WOG) and extremely preterm birth (<28 WOG), or with regard to birth weight, into low (LBW; <2500g), very low (VLBW; <1500g) and extremely low birth weight (ELBW; <1000g) [1, 2]. The increasing incidence of preterm birth among live births is 11% worldwide, ranging from 5% in parts of Europe to 18% in parts of Africa [3, 4]. Since the child's organs are not fully developed, prematurity is associated with numerous complications that often lead to permanent damage and lifelong impairments, resulting in significantly increased morbidity and mortality rates [3].

The combination of undeveloped organs and the high need for invasive care and life support measures is of great importance in the pathogenesis of many complications including infections [5]. In particular, the immature immune system plays an important role: numerous factors of the innate and acquired immune system are demonstrably suppressed and limited in their development and function, which explains the susceptibility of preterm infants to infectious diseases [6, 7]

Several studies have shown that the risk of infection increases with decreasing gestational age and birth weight [8, 9]. Preterm infants are at a significantly increased risk of suffering serious infections that require hospitalisation and treatment [10]. The entities responsible for the increased risk of infection in preterm infants are neonatal sepsis and respiratory infections, with respiratory tract infections caused by respiratory syncytial virus (RSV) in particular [11].

The group of late preterms is an extremely important subgroup, accounting for almost 75% of all preterm infants and numerically the most fasting growing subgroup of newborns [12]. Although the late preterms are often treated as full-term infants in the clinical setting, they have significantly higher morbidity and mortality rates compared to full-term infants [12, 13]. Nevertheless, they are often discharged inappropriately early after birth, resulting in a significantly higher rehospitalisation rate and the associated higher health care costs.

The overall aim of this monocentric prospective observational study was to investigate whether preterm infants were more likely to suffer from infections than full-term infants during their first year of life, what types of infections were present and whether associated factors were observable.

## Methods

Ethical approval for this monocentric prospective observational study was applied for and granted by the ethics committee of the Medical University of Vienna (EK-Nr: 1330/2016). Participants were recruited in a Viennese pediatric practice, the “First Vienna Pediatric Medical Center”. Exact case number planning was not required due to the exploratory approach of this study. Parents were informed about the study and gave written consent. Data of infants were pseudonymized for analysis.

Inclusion criteria for the observation group of preterm infants was a birth before WOG<37+0, and for full-term group a birth after WOG >37+0. Gestational age was based on parental report and calculation according to a gynaecologist. In case of inconsistent results of sonography and calculated gestational age, adaptions were done by the gynaecologist. Children with chronic disease were excluded from the study. Those infants, who were primarily recruited, but did not appear in the practice during the observation period were finally excluded from the study analysis as drop out. Data were analysed only from those infants, who completed the observation period of one year. The general, demographic and birth-specific data were collected by the legal guardians using a questionnaire.

Subsequently, study subjects were asked to attend the study center in case of infections. They were observed throughout the first year of life for infections and complications that occurred since birth, which was documented in the patient records. At the end of the first year of life, the total number of infections and the number of different types of infection were calculated for each child. The different types of infection included respiratory, otorhinolaryngeal (ENT-infections), urinary tract (UT- infections), gastrointestinal (GI-infections), dermal and severe infections. Infections that could not be assigned to any of these groups were assigned to the category of *other infections*. Serious infections were thus defined as those that led to hospitalisation.

## Statistics

The raw data (S1 Table) was compiled in Microsoft Excel and analysed using IBM SPSS-Statistics (SPSS 22 for Mac OSX, version 22.0) and graph pad prism software (Prism 6 for MacOS X). Missing data was excluded from the calculation and statistical significance level was set at p<0.05.

To estimate the practical relevance of statistically significant relations, the standardised effect size *r* was used according to the Cohen classification (*r* = .10-.29 minor, *r* = .30-.49 moderate and *r*≥ .50 great effect) [14]. The odds ratio (OR) was considered as an effect measure for the relative risk and 95% confidence interval (CI) was reported. For analysis of differences of nominally scaled variables, chi-square test (X^2^) or in cases of expected values <5 in more than 20% of the cells, Fishers Exact (cF) test were performed. The McNemar test was used to test relationships of dependent nominally scaled variables. For analysis of strength and direction of association of two ranked, non-parametric variables, Spearman correlation coefficient (r_s_) was calculated. Differences of two independent samples were tested either with t-test (for total number of infections) or the Mann Whitney U-test as a parameter-free alternative. Kruskal-Wallis test (H) was performed for comparison of medians of more than 2 groups. Multiple linear regression was performed for analysing the influence of diverse predictors. Two-way ANOVA was used for testing for differences of infection frequency per month of age. Correction of multiple testing was done using the Holm-Bonferroni method and adjusted p-values were calculated.

## Results

Eighty preterm and eighty term infants were primarily recruited in this study. Due to loss of follow up, being originated in changing the paediatrician during the observation period, the original case count was reduced to 71 for the full-term and 72 for the preterm infants, which corresponds to a drop-out rate of 10.6%.

As depicted in Table 1, the sample included 55 (76.4%) late, 13 (18.1%) very and 4 (5.6%) extremely preterms, 25 (34.7%) of all preterm infants were included in the age-specific normal weight group, 32 (44.4%) in the LBW, 10 (13.9%) in the VLBW and 5 (6.9%) in the ELBW group.

**Table 1:** Baseline characteristics.

	full-term infants (WOG ≥ 37+0) n = 71	preterm infants (WOG< 36+6) n = 72	test statistic	test parameter (df)	*p*	sig.	*p*.adjust
**Sex**^**2**^							
Male	40 (54.1%)	34 (45.9%)	X^2^	1.19 (1)	.275	n.s.	1.
Female	31 (44.9%)	38 (55.1%)					
**birth mode**^3^							
natural	51 (71.8%)	33 (45.8%)	X^2^	9.96 (1)	.002	**	.028*
Cesarean	20 (28.2%)	39 (54.2%)					
**seasonal birth time date**							
warm season	46 (47.9%)	50 (52.1%)	X^2^	0.35 (1)	.553	n.s.	1.
cold season	25 (53.2%)	22 (46.8%)					
**number of siblings**	0.66 ± 0.88	0.92 ± 1.02	*U-*test (*z*)	-1.66	.096	°	.96
**pregnancy- and birth complications**^3^							
None	54 (76.1%)	46 (63.9%)	X^2^	12.44 (1)	< .001	**	.018*
Occurred	17 (23.9%)	26 (36.1%)					
**perinatal complications**^3^							
None	37 (52.1%)	13 (18.1%)	X^2^	8.51 (1)	.004	**	.048*
Occurred	34 (47.9%)	59 (81.9%)					
**art of daycare**^3^							
at home	64 (90.1%)	65 (90.3%)	X^2^	0.01 (1)	.978	n.s.	1.
Nursery	7 (9.9%)	7 (9.7%)					
**living situation**^3^							
House	17 (23.9%)	19 (26.4%)	X^2^	0.53^1^	.885	n.s.	1.
Flat	52 (73.2%)	52 (72.2%)					
smoking home	2 (2.8%)	1 (1.4%)					
**breast feeding**^3^							
No	18 (25.24%)	20 (27.8%)	X^2^	0.11 (1)	.743	n.s.	1.
Yes	53 (74.6%)	52 (72.7%)					
**length of postnatal hospital stay**	5.18 ± 1.16	27.52 ± 32.57	*U-*test (*z*)	-7.95	< .001	**	.018*
**total number of infections**	3.85 ± 1.72	6.01 ± 3.90	t-test	32.44^1^	< .001	**	.018*
number of respiratory infections	1.03 ± 1.06	1.82 ± 1.71	*U-*test (*z*)	-2.92	.003	**	.039*
number of ORL infections	2.00 ± 1.30	2.72 ± 2.22	*z*	-1.46	.144	n.s.	1.
number of GI infections	0.15 ± .44	0.17 ± .41	*z*	-.40	.687	n.s.	1.
number of urinary tract infections	-	0.04 ± .20	*z*	-1.73	.083	°	.913
number of dermal infections	0.14 ±.35	0.14 ± .42	*z*	-.46	.643	n.s.	1.
number of other infections	0.44 ± .67	0.57 ±.71	z	-1.21	.227	n.s.	1.
number of severe infections	0.08 ± .28	0.56 ± 1.10	z	-3.63	< .001	**	.018*

Characteristic values (mean value and standard deviation for metric values as well as frequencies and proportions for categorical parameters) of the study-relevant parameters as a function of the maturity status with test size and significance assessment.

** p ≤.01

* p ≤.05

° p ≤.10 (tendency)

^1^corrected by exact Fisher test (cF); for better readability, mean values and standard deviations were used for certain X^2^ and U tests and t-test

^2^Line percentages

^3^Column percentages.

P.adjust: adjusted P-value after Holm-Bonferroni correction for multiple testing.

There was an even gender distribution with 40 (54.1%) male and 31 (44.9%) female full-term infants and 34 (45.9%) male and 38 (55.1%) female preterm infants.

With regard to the birth procedure, significantly more preterms (54.2%) were born by Caesarean section (X^2^(1) = 9.96 and *p* = .002) compared to term infants (28.2%) indicating a threefold higher rate of being born by caesarean section compared to full-term infants (OR 3.01 [95%CI 1.51; 6.04]).

Concerning pregnancy and birth complications, 36.1% of preterm and 23.9% of full-term infants showed a complication significantly associated with preterm birth (X^2^(1) = 12.444, *p* < .001). Nuchal cord as most common complication in term infants (7%), leading to intrauterine asphyxia in two cases, was followed by gestational diabetes (GDM) with concomitant macrosomia, isolated GDM, polyhydramnios and premature rupture of the membranes (PROM) (1.4% each)). In the preterm infants group, the most frequent complications were pre-eclampsia (9.7%), preterm rupture of membranes (9.7%), maternal HELLP syndrome (5.5%) and GDM (5.5%). Severe preeclampsia in combination with placental insufficiency occurred with 2.8%, and placenta praevia and premature placental abruption with 1.4%.

Perinatal complications were observed in 81.9% of preterm infants and 47.9% of full-term infants with a significantly higher rate in preterm infants (X^2^(1) = 8.511 and *p* = .004) with about five times greater risk (OR = 4.94, [95% CI 2.31; 10.56]). In preterm infants, 28 (38.9%) had respiratory distress syndrome, 2.8% intraventricular hemorrhage, 1.4% necrotizing enterocolitis, 9.7% patent ductus arteriosus and 4.2% a retinopathy of prematurity. Twelve (16.7%) suffered from a congenital infection, 1.4% had neonatal sepsis 55.5% had neonatal jaundice. Thirty-three (46.5%) full-term infants had neonatal jaundice and one of them each had a congenital infection and neonatal sepsis (1.4%).

As for the number of siblings, a trend could be assumed that preterm infants with a mean of 0.92 (min 0, max 5) had a higher number of siblings than full-term infants with a mean value of 0.66 (min 0, max 3) (*U* = 2175.5 (-1.664), *p* = .096).

With regard to the form of care, 64 (90.1%) full term infants were looked after at home and seven (9.9%) in a nursery, whereas within the preterms 65 (90.3%) grew up at home and seven (9.7%) in a nursery. As for the housing situation, 19 (26.4%) preterms grew up in a house, 52 (72.2%) in an apartment and one (1.4%) in a smoking household. Regarding the full-term infants 17 (23.9%) grew up in a house, 52 (73.2%) in an apartment and two (2.8%) in a smoking household. As for the breastfeeding factor, preterm infants (72.7%) and full-term infants (74.6%) were breastfed equally frequently.

In the present sample, it could be shown that preterm infants had a significantly longer hospital stay after birth compared to full-term infants (*U* = 858.0 (z = -7.951), *p* < .001, *r* = .67), with an average duration of 5.18 days for full-term and 27.52 days for preterm infants.

**Fig 1:**
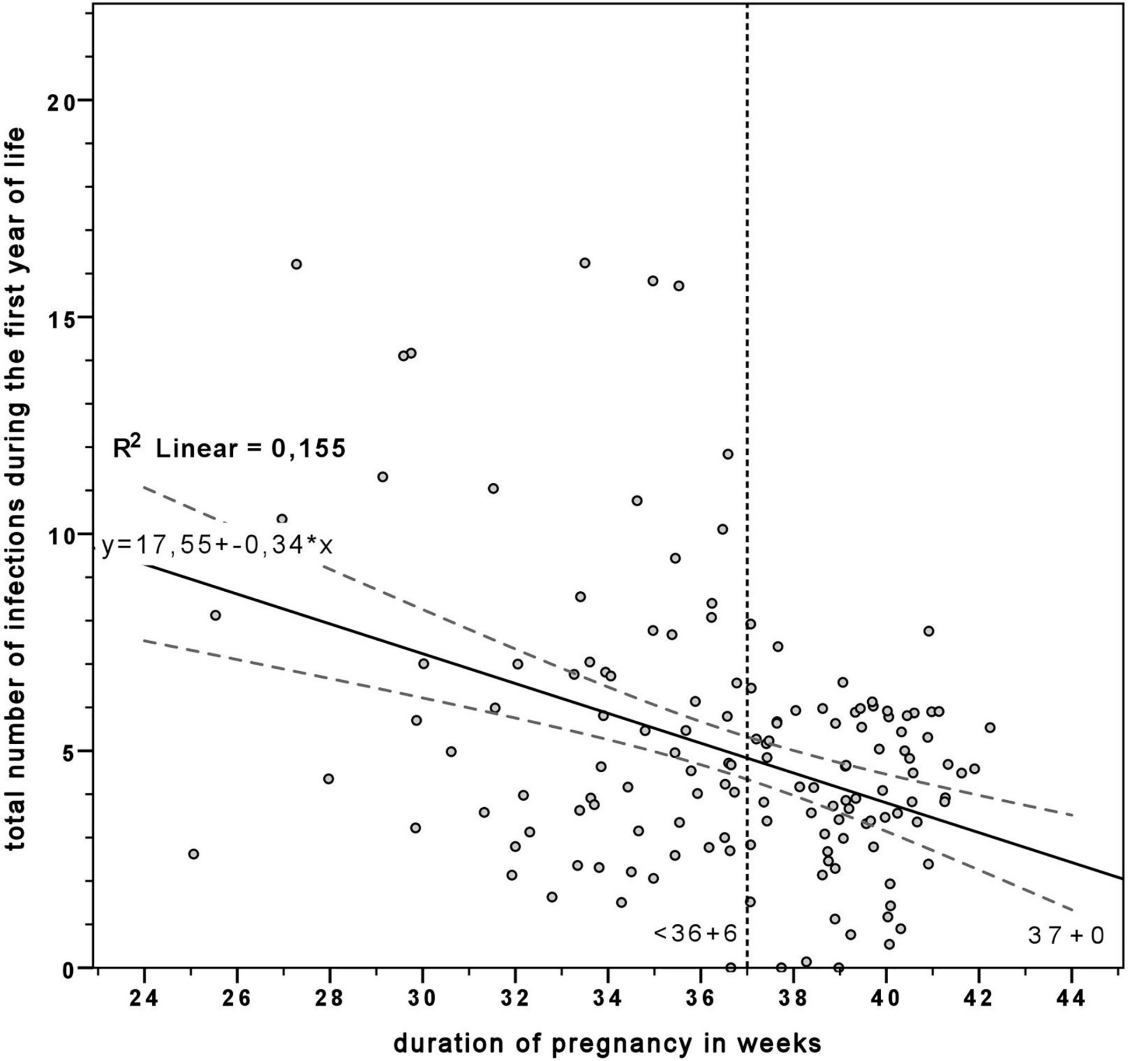
Higher risk of infection in preterm infants. Moderate, but significantly negative correlation between the maturity status and the risk of infection tested by linear regression and shown by bivariate scatter plot graph.

Preterm infants had a significantly higher total number of infections within the first year of life than full-term infants (*p* < .001). In full-term infants, 3.85 ±1.72 (min 0, max 7, *Md* = 4.0), and in preterm infants 6.01 ±3.90 (min 0, max 17, *Md* = 5.0) infections were detected during the first year of life. The coincidence was tested by a bivariate plot graph (Fig 1) and showed a moderate, but significantly negative correlation between the maturity status and the risk of infection (*r*(143) = -.394, *p* < .001).

Concerning the group of preterm infants, it was investigated whether there is a difference in the risk of infection with regard to gestational age and birth weight. Both analyses showed a non-significant result (gestational age: X^2^(2) = 3.193, *p* = .203; birth weight: X^2^(3) = 1.766, *p* = .622) (Table 2).

**Table 2:** Total number of infections in preterm infant group.

	n = 72	test statistic	*test parameter (df)*	*P*	sig.
**gestational age**					
late preterm	5.56 ± 3.58				
very preterm	6.77 ± 4.34	***H***	3.19 (2)	.203	n.s.
extremely preterm	9.75 ± 5.38				
**birth weight**					
normal birth weight	6.04 ± 3.88				
low birth weight	5.28 ± 3.23	***H***	1.77 (3)	.622	n.s.
very low birth weight	7.30 ± 5.08				
extremely low birth weight	8.00 ± 4.90				

Mean value, standard deviation, test size and significance assessment of the total number of infections within preterm infants with regard to gestational age and birth weight

**Fig 2:**
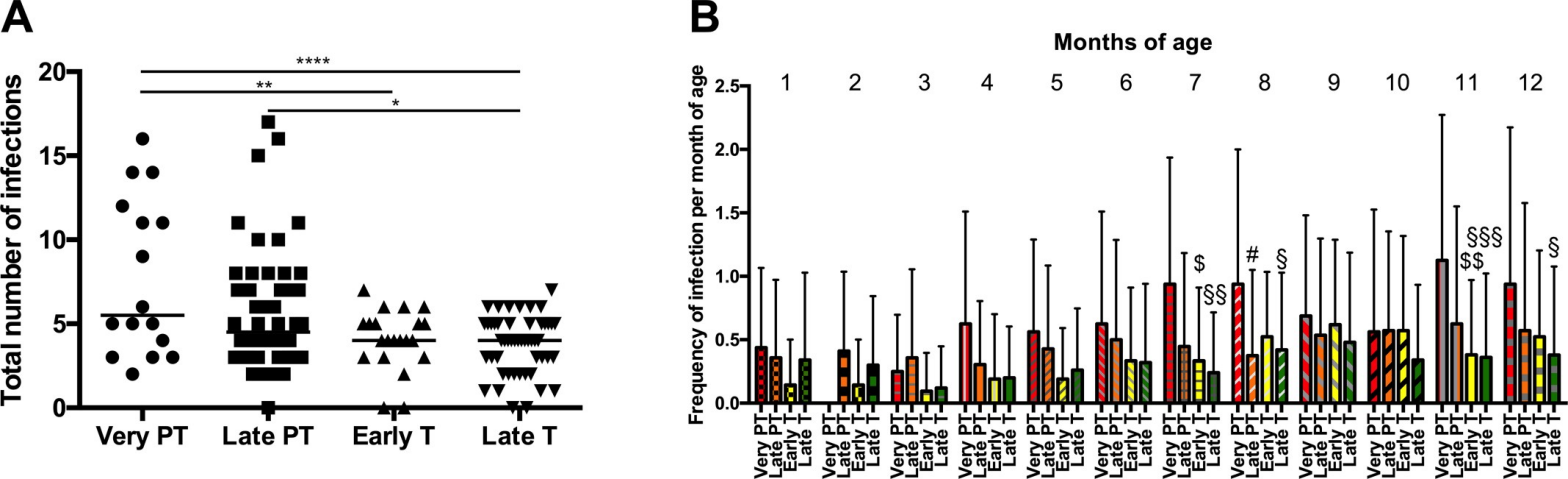
Number of infections in very preterm, late preterm, early term and late term infants. Infection number of respective groups within the first year are depicted in A. Frequency of infections are shown for each month of age for each group. PT: preterm, T: term, *adjusted p<0.05, ** adjusted p<0.01. ****adjusted p<0.0001. $ (very PT-early T): p<0.05, $ $ (very PT-early T): p<0.01, § (very PT-late T): p<0.05, §§ (very PT-late T): p<0.01, §§§ (very PT-late T) p<0.001, # (very PT-late PT): p<0.05.

Extremely and very preterm infants did not differ regarding the number of infections, which is the reason for combining these groups for the next analysis. As gestational age might be only estimated within 1–2 weeks, the full-term babies were divided into early term infants (WOG 37+0 to 38+6) and late term (> = 39+0 WOG). The total frequency of infections within the first year of life was compared between very preterm babies (<32 WOG, n = 16), late preterm infants (32+0 to 36+6 WOG, n = 56), early term (n = 21) and late term infants (n = 50) (Fig 2). No significant differences were observed between early term and late term infants (adjusted p>0.9999). The very preterm group had significantly more infections than the early and late term group (adjusted p = 0.0019, and adjusted p = <0.0001). The late preterm and late term groups differed significantly in total number of infections (adjusted p = 0.0154). The frequency of infections per month of age was calculated for these groups and revealed by trend more infections at the end of the first year of life and significantly differences at the age of 8, 11 and 12 months of age between very early preterm and term infants (Fig 2). The relative risk for preterm babies to have more than 1 infection in the first year of life is significantly higher compared to term babies (OR 9.016, (95%CI 1.096 to 74.13), *p = 0.0174).

With regard to the different types of infection, it was found that respiratory (z = -2.923, p = .003, r = .24) and severe (z = -3.633, p < .001, r = .30) infections showed a significant difference in the comparison of the two observation groups. The most frequent severe infections leading to hospitalization were, except for connatal infections directly after birth, respiratory infections (42%), gastroenteritis (33%), fever of unknown origin (9%), urinary tract infection, influenza with emesis and meningitis (each 4%).

For the group of UTI, the quantity tested (p = .083, r = .14) was significant, although only 3 preterm and no term infants suffered from UT-infections (Table 3).

**Table 3:** Type of infections in preterm and full-term infants.

	preterm (n = 72) WOG ≤36+6			full-term (n = 71) WOG > 37+0		
art of infection	*M* ± *SD*	*Md*	min; max	*M* ± *SD*	*Md*	min; max
respiratory infections	1.82 ± 1.71	1.0	0; 9	1.03 ± 1.06	1.0	0; 4
otorhinolaryngeal infections	2.72 ± 2.22	2.0	0; 11	2.00 ± 1.30	2.0	0; 5
gastrointestinal infections	0.17 ± .41	0	0; 2	0.15 ± .44	0	0; 2
urinary tract infections	0.04 ± .20	0	0; 1	-	-	-
dermal infections	0.14 ± .42	0	0; 2	0.14 ± .35	0	0; 1
other infections	0.57 ± .71	0	0; 2	0.44 ± .67	0	0; 3
severe infections	0.56 ± 1.10	0	0; 7	0.08 ± .28	0	0; 1

Mean and standard deviation, medium, min and max are depicted.

As possible predictors that can influence the total number of infections, the following additional factors were checked: gender, birth procedure (cesarean or natural birth), birth in the warm (March-September) vs. cold season (September-March), number of siblings, presence of pregnancy and childbirth complications, occurrence of perinatal complications, child care, living conditions and breastfeeding during the first year of life. The analysis showed that only the duration of pregnancy (*p* = .002) as well as the number of siblings (*p* = .038) had a significant influence. The individual predictors were also analysed separately for their influence on the risk of infection, which in turn showed that there was a weak positive relationship between number of siblings and total number of infections (*rs*(143).235, *p* = .002). A weak positive relationship between hospital stay after birth in days and the total number of infections during the first year of life were found (*rs*(142).17, *p* = .044). The analysis of the remaining predictors revealed insignificant effects.

## Discussion

The pathogenesis and therapy of perinatal complications of preterm birth are subjects of research. Few studies dealt with the consequences of preterm birth, which extend beyond childhood. Preterm study participants who fail to comply or discontinue their participation in a longitudinal study may lead to bias. This is also to be cited as a limiting factor in this study, which has a dropout rate of 10.6% mainly due to the change of paediatrician.

In the present study, the numbers of preterm infants are close to data from literature, which in some cases vary widely in different regions of the world [15]. The comparatively small sample size—above all the low proportion of the very and extremely preterms—represents a limiting factor in this study, as a result of which only significant effects led to a significant difference and presumable differences based on existing literature could probably not be revealed.

In the sample concerned, there was a significantly higher total number of infections in preterm compared to full-term infants. In the only comparable study, the total number of infectious diseases in the first 12 months of life were 0–13 (*Md* = 4.0) in preterm and 0–5 (*Md* = 2.0) in full-term infants. The most frequent reasons for consultation were bronchitis, rhinopharyngitis, otitis and gastroenteritis, while in the group of preterm infants, more severe infections, febrile convulsions and breath-holding spells were observed [16]. In line with these results, ENT infections followed by respiratory infections occurred most frequently in both our groups, while preterm infants additionally had a significantly higher number of serious infections, mainly gastroenteritis and respiratory infections leading to hospitalization. In recent literature, the increased respiratory infection rates of preterm infants are discussed, rather than the total number of infections and the incidence of the different types of infection. This could be due to the fact that respiratory tract infections are among the most common childhood infections [17]. Besides, the relatively frequently occurring perinatal respiratory complications and the associated long-term consequences in preterm infancy could play a role. In a Swedish study, the bronchiolitis rate in the first year of life as well as the rate of hospital stay were significantly higher in terms of a serious infection in preterm infants than in full-term infants [18]. Preterm infants, in particular, are more likely to suffer from severe forms of RS virus bronchiolitis requiring hospitalization [19].

The only factors that had a significant impact on total number of infections in this study included pregnancy duration, sibling numbers, and duration of postnatal hospital stay. Recent publications reported that the care in a nursery, as well as the presence of siblings have a significant influence on the risk of infection [17, 20, 21] due to the early contact with their elder siblings’ infections. Recent reports showed an association of respiratory infections in infants exposed to tobacco smoke [22], which was beyond the scope of our study to be analysed sufficiently. The described weak positive association between postnatal hospital stay and the increased risk of infection could possibly be due to the high microbial load in the hospital and the particularly high vulnerability of preterm infants to nosocomial infections [23], which is in compliance with other publications [13]. Whether the breastfeeding factor actually has a protective effect on the risk of infection in infants remains controversial [20]. In accordance with our results, it was observed in a German cohort study that the time of birth does not influence the incidence of infections [17].

In the respective sample, no difference in the total number of infections could be detected in the different age and weight classes of preterm infants, which is probably attributable to the comparatively small sample size. Based on previous findings, an inverse proportional relation would be expected between risk of infection and the gestational age and birth weight [24, 25]. Additionally, no differences between early term and late term babies were found, which is of importance as the gestational age might be only estimated within 1–2 weeks. On the other hand, there was a clear association between the maturity status in terms of preterm birth and the occurrence of pregnancy, birth and perinatal complications. The complication rates in the sample concerned were comparable to those described in literature [3, 26].

The limiting factors of this study are mainly the small sample size and the possibility of incomplete data. During the study, it was requested that the study participants would always visit the pediatric practice if they suffered from infections. The possibility of visiting another doctor or not mentioning an infection that has been treated elsewhere (for example, on an inpatient basis) could have falsified the results.

## Conclusion

Preterm infants represent a particularly significant and vulnerable group with increasing survival rates. Knowing the higher risk of complications and infections in preterm infants during their first year of life, more comprehensive care should be offered to these children. The care of preterm infants should not end after discharging them and, above all, late-preterm infants should not be mistakenly regarded as full-terms and treated as such. Parents should be informed about the increased risk of infection and the preventive measures urgently. Vaccination of all family members who come into contact with the infant, adequate hand disinfection and avoidance of environments that increase the microbial load are important prophylactic measures.

## Supporting information

S1 Table Dataset of complete raw data for statistical analysis included as SPSS file. (SAV)

## Abbreviations

X2: Chi-Square test
c.F.: Fisher’s exact test
df: degrees of freedom
EK-Nr.: Ethics committee registration number
ELBW: Extremely low birth weight
ENT-infections: Ear nose throat infections
GDM: Gestational diabetes mellitus
GI-infections: Gastrointestinal infection
HELLP-syndrome: Haemolysis Elevated Liver enzymes Low Platelet count-syndrome
IVH: Intraventricular haemorrhage
CI: Confidence Interval
LBW: Low birth weight
M: Mean value
Md: Median
NEC: Necrotizing enterocolitis
n.s.: not significant
OR: Odds Ratio
PDA: Patent ductus arteriosus
PROM: Premature rupture of membranes
PT: Preterm
RDS: Respiratory distress syndrome
ROP: Retinopathy of prematurity
RS-virus: Respiratory Syncytial Virus
Sig.: Significant
T: Term
UT-infections: Urinary tract infection
VLBW: Very low birth weight
WOG: Week of gestation
